# Circulating N-Acetylaspartate does not track brain NAA concentrations, cognitive function or features of small vessel disease in humans

**DOI:** 10.1038/s41598-022-15670-0

**Published:** 2022-07-07

**Authors:** Eleni Rebelos, Giuseppe Daniele, Beatrice Campi, Alessandro Saba, Kalle Koskensalo, Jukka Ihalainen, Ekaterina Saukko, Pirjo Nuutila, Walter H. Backes, Jacobus F. A. Jansen, Pieter C. Dagnelie, Sebastian Köhler, Bastiaan E. de Galan, Thomas T. van Sloten, Coen D. A. Stehouwer, Ele Ferrannini

**Affiliations:** 1 Institute of Clinical Physiology, National Research Council (CNR), Via Savi, 10, 56126 Pisa, Italy; 2 Turku PET Centre, University of Turku, Turku, Finland; 3 Department of Clinical and Experimental Medicine, University of Pisa, Pisa, Italy; 4 Department of Surgical, Medical and Molecular Pathology and Critical Care Medicine, University of Pisa, Pisa, Italy; 5 Laboratory of Clinical Pathology, St. Chiara University Hospital, Pisa, Italy; 6 Department of Medical Physics, Turku University Hospital, Turku, Finland; 7 Department of Radiology, Turku University Hospital, Turku, Finland; 8 Department of Medicine, Maastricht University Medical Centre, Maastricht, The Netherlands; 9 Cardiovascular Research Institute Maastricht (CARIM), Maastricht University Medical Centre, Maastricht, The Netherlands; 10 Department of Radiology and Nuclear Medicine, Maastricht University Medical Centre, Maastricht, The Netherlands; 11 School for Mental Health and Neuroscience (MHeNs), Maastricht University Medical Centre, Maastricht, The Netherlands; 12 Alzheimer Center Limburg, Maastricht University Medical Center, Maastricht, The Netherlands

**Keywords:** Cognitive neuroscience, Neural ageing, Medical research

## Abstract

N-acetylaspartate (NAA) is the second most abundant metabolite in the human brain; although it is assumed to be a proxy for a neuronal marker, its function is not fully elucidated. NAA is also detectable in plasma, but its relation to cerebral NAA levels, cognitive performance, or features of cerebral disease has not been investigated. To study whether circulating NAA tracks cerebral NAA levels, and whether circulating NAA correlates with cognitive function and features of cerebral small vessel disease (SVD). Two datasets were analyzed. In *dataset 1*, structural MRI was acquired in 533 subjects to assess four features of cerebral SVD. Cognitive function was evaluated with standardized test scores (*N* = 824). In *dataset 2*, brain ^1^H-MRS from the occipital region was acquired (*N* = 49). In all subjects, fasting circulating NAA was measured with mass spectrometry. *Dataset 1:* in univariate and adjusted for confounders models, we found no correlation between circulating NAA and the examined features of cerebral SVD. In univariate analysis, circulating NAA levels were associated inversely with the speed in information processing and the executive function score, however these associations were lost after accounting for confounders. In line with the negative findings of *dataset 1*, in *dataset 2* there was no correlation between circulating and central NAA or total NAA levels. This study indicates that circulating NAA levels do not reflect central (occipital) NAA levels, cognitive function, or cerebral small vessel disease in man.

## Introduction

One of the most abundant metabolites in the mammalian brain is N-acetylaspartate (NAA), an aminoacid with concentrations in the central nervous system (CNS) in the millimolar range (~ 10 mM)^1^. NAA derives from L-aspartic acid, synthesized in neuronal mitochondria from acetyl-coenzyme A. The two-carbon acetate portions of the latter derive predominantly from the oxidation of glucose. Even though NAA is synthesized and stored primarily in neurons, it cannot be catabolized by them^2^ and is therefore released into the extracellular fluid (ECF). Following diffusion to oligodendrocytes, aspartoacylase (ASPA) cleaves the acetate moiety and aspartate is recycled back to neurons^3^. N-acetyl aspartate glutamate (NAAG) is also synthesized in neurons and is converted to NAA in astrocytes.

Although the function of NAA in humans remains to be fully elucidated^4^, it has been proposed that one of its most important functions is acting as a neuronal molecular water pump where each NAA molecule with a minimum of 32 water molecules^5^ is released by neurons to the ECF down a steep NAA gradient, effectively transporting water to ECF. NAA may also act as a facilitator of energy metabolism and as a source of acetate for fatty acid and steroid synthesis necessary for axonal myelination by oligodendrocytes^4^.

Thanks to the abundant presence of NAA in neurons and its *N*-acetyl methyl group resonance, NAA and total NAA (i.e. NAA + NAAG) can be detected with brain ^1^H magnetic resonance spectroscopy (MRS). NAA is considered to be a proxy marker of neuronal health^6^; indeed brain spectroscopy studies have shown NAA deficits in normal aging^7^ and in several neurological disorders such as in Alzheimer’s disease^8–10^, Huntington disease^11,12^, and multiple sclerosis^13,14^, and in psychiatric diseases such as bipolar disorders^15–17^, and schizophrenia^16,18,19^. A recent meta-analysis showed that presence of type 2 diabetes (T2D) is also associated with decreased levels of NAA in the frontal lobe and the lenticular nucleus, while no significant changes in NAA levels were found in the occipital and parietal lobes and in thalamus^20^. At the other end of the spectrum, excessive NAA accumulation caused by a missense mutation in the *ASPA* gene results in a fatal neurodegenerative disorder, the Canavan disease, characterized by widespread CNS vacuolization and hypomyelination^21^.

Neuronal NAA metabolism is dynamic with a turnover rate of ~ 1.4 times/day^1^. Also, there are now several lines of evidence showing that neuronal stimulation or specific treatments impact on central NAA concentrations. For instance, a study has shown that during periods of visual stimulation, NAA concentrations in the visual cortex are decreased, whereas upon stimulus cessation, central NAA concentrations are restored^22^. This dynamic modification of central NAA concentrations upon stimulation and stimulus withdrawal has been suggested to indicate an intimate association of NAA metabolism and neurostimulation^22^. In the same line, treatment with levodopa in patients with Parkinsons’ disease^23^, antidepressant medication in depressed patients^24^, or alcohol withdrawal in patients with alcohol abuse^25^ have all been shown to restore central NAA levels.

Previous studies have linked central NAA concentrations with cognition^26^, even though the available literature is not unanimous on the topic. For instance, even though reductions in central NAA concentrations have been linked to worse cognitive function, this correlation is rather weak^27^, and other studies have failed to detect any association between cognition and NAA concentrations^28^. To some extent the discrepancy in the existing literature could be attributed to varying portions of gray and white matter inside the sampling MRS voxels, thereby affecting the concentrations of the assessed metabolites^29^ and consequently also their associations to cognitive parameters. Also, it is be reasonable to expect that correlations between neurometabolites and cognitive function might vary according to the cortical region investigated and its functional specialization.

Cerebral small vessel disease (SVD) is an “umbrella term” encompassing a variety of abnormalities related to cerebral microangiopathy^30^. It is a major cause of cognitive impairment and dementia^31^. Because of the mild clinical symptoms and the low case fatality rate of cerebral SVD, it is often discovered as incidental findings in neuroimaging studies, and its prevalence increases with advancing age^32^. Evidence suggests that patients with cerebral SVD have lower brain NAA concentrations compared to healthy controls, and that central NAA concentrations correlate negatively with the lesion volume^28^.

NAA is also detectable in the plasma, and ASPA is abundantly expressed in peripheral organs in addition to oligodendrocytes^33^. However, to the best of our knowledge whether circulating NAA tracks central NAA levels has not been investigated. Moreover, whereas previous studies have linked central NAA concentrations to both cognitive function^26^ and cerebral SVD^28^, it is not known whether circulating NAA concentrations associate with cognitive function or with cerebral SVD. Thus, the primary aim in the present study was to assess the value of measuring plasma NAA concentrations and their relation to features of cognitive dysfunction and cerebral small vessel disease. Also, in a separate smaller study we assessed the interplay between central (occipital) and circulating NAA concentrations.

## Materials and methods

### Study subjects

In this study we combined data from two different datasets: *dataset 1* is a random subset of The Maastricht Study^34^; *dataset 2* is from a clinical trial (performed at the Turku PET Centre, NCT04343469) whose main aim was to study brain inflammation in human obesity. The Maastricht Study is an observational prospective population-based cohort study. The rationale and methodology have been described previously^34^. In brief, the study focuses on the etiology, pathophysiology, complications and comorbidities of type 2 diabetes mellitus (T2D) and is characterized by an extensive phenotyping approach. Eligible for participation were all individuals aged between 40 and 75 years and living in the southern part of the Netherlands. Participants were recruited through mass media campaigns and from the municipal registries and the regional Diabetes Patient Registry via mailings. Recruitment was stratified according to known T2D status, with an oversampling of individuals with T2D, for reasons of efficiency. The examinations of each participant were performed within a time window of three months. The study has been approved by the institutional medical ethical committee (NL31329.068.10) and the Minister of Health, Welfare and Sports of the Netherlands (Permit 131088-105234-PG). All participants gave written informed consent. In The Maastricht Study (*Dataset 1, n* = *824),* participants had a thorough evaluation of their cognitive function (three cognitive domains were explored: verbal memory, processing speed and executive function), and 533 of them also underwent a structural brain MRI for the evaluation of cerebral small vessel disease features. Participants in *dataset 2* underwent brain ^1^H-MRS in the fasting state (n = 49). All subjects had fasting circulating NAA measured after an overnight fast. In *dataset 1* mean age was 60 ± 8 years, mean BMI was 27.1 ± 4.5 kg/m^2^, ~ 30% of the population had T2D and ~ 80% of the population had prior cardiovascular disease. In *dataset 2,* the study participants (45 ± 9 years of age) included morbidly obese individuals (N = 25) and their lean controls (N = 24), none of whom had prior cardiovascular disease. The characteristics of the study participants are given in Tables 1 and 2. The study protocols were approved by the local ethics committee of Maastricht University Medical Centre (NL31329.068.10) and the Ministry of Health, Welfare, and Sports of the Netherlands (Permit 131088-105234-PG), and the local ethics committee of Turku University Hospital, respectively. Written informed consent was obtained from all volunteers before their participation in the studies. In both studies, all procedures performed were in accordance with the Helsinki Declaration.

**Table 1:** Clinical characteristics of *Dataset 1* according to median NAA value (70.4 ng/mL).

	Lower than median NAA value (n = 413)	Higher than median NAA value (n = 411)
Age, mean years (SD)	59 ± 9	61 ± 8
Men, %	56.7	43.1
**Education**		
Low, %	31.2	34.1
Intermediate, %	31.0	28.0
High, %	37.8	38.0
**Glucose metabolism status**		
Normal glucose metabolism, %	47.2	58.4
Prediabetes, %	16.5	15.8
Type 2 diabetes, %	35.1	25.1
Other type diabetes, %	1.2	0.7
HbA_1c_ (%)	6.1 ± 1.1	5.8 ± 0.8
Body mass index, mean kg/m^2^ (SD)	27.6 ± 4.3	26.9 ± 4.5
Current smokers, %	32.7	34.3
Systolic blood pressure, mean mmHg (SD)	136 ± 18	133 ± 18
Diastolic blood pressure, mean mmHg (SD)	77 ± 10	76 ± 10
Hypertension, %	61.0	52.4
Antihypertensive medication use, %	43.4	36.3
Prior cardiovascular disease, %	83.3	85.2
Features of cerebral small vessel disease*		
Total brain parenchyma volume, mean ml (SD)	1142 ± 111	1131 ± 112
White matter hyperintensity volume, median ml (interquartile range)	0.2 [0.1–0.6]	0.2 [0.1–0.8]
Cerebral microbleeds, %	88.6	87.3
Lacunar infarcts, %	6.9	5.7
**Cognitive function**		
Verbal memory, mean Z-score (SD)	0.15 ± 0.89	0.12 ± 0.91
Information processing speed, mean Z-score (SD)	0.05 ± 0.73	0.01 ± 0.75
Executive function, mean Z-score (SD)	0.11 ± 0.72	0.10 ± 0.76

* Data available in n=533.

**Table 2:** Clinical characteristics of *Dataset 2*.

	Obese	Lean	*p* value
Men/Women	7/18	9/15	ns
Age (years)	47 ± 10	43 ± 9	ns
NGT/IFG&IGT/T2D	10/9/6	23/1/0	0.007
BMI (kg/m^2^)	40.7 ± 5.3	24.9 ± 3.2	< 0.0001
Waist/Hip	0.97 ± 0.12	0.87 ± 0.08	0.002
HbA_1c_ (%)	5.6 ± 0.5	5.2 ± 0.4	0.0006
MAP (mmHg)	103 ± 11	90 ± 9	0.0005
Total cholesterol (mmol/L)	4.3 ± 0.8	4.2 ± 0.7	ns
LDL cholesterol (mmol/L)	2.6 ± 0.6	2.6 ± 0.9	ns
HDL cholesterol (mmol/L)	1.2 ± 0.2	1.5 ± 0.4	0.01
Triglycerides (mmol/L)	1.4 ± 0.5	1.0 ± 0.5	0.01
Fasting plasma glucose (mmol/L)	5.7 ± 0.8	5.0 ± 0.4	0.003
Fasting plasma insulin (pmol/L)	110 ± 72	45 ± 24	< 0.0001
C-reactive protein (mg/dL)	3.24 ± 2.12	0.67 ± 0.65	< 0.0001
Circulating NAA (ng/mL)	65.4 [11.6]	62.8 [12.1]	ns
Central NAA (mmol/L)	9.1 [0.4]	9.6 [0.7]	0.001
Central TNAA (mmol/L)	9.4 [0.7]	10.0 [0.7]	0.004

Entries are mean ± SD or median [IQR].

### Brain MRI and ^1^H-MRS protocols

**Figure 1:**
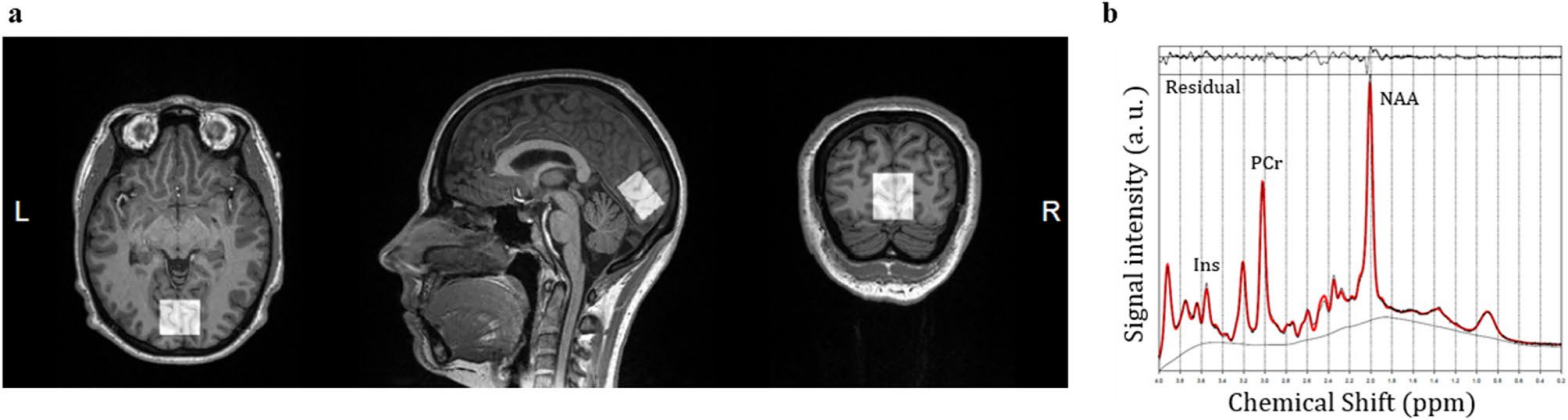
Voxel placement for ^1^H MRS in the occipital lobe (**a**), and a representative example of a fitted spectrum (**b**).

In *dataset 1*, brain MRI was performed on a 3 T MRI scanner (Siemens Magnetom Prisma-fit Syngo MR D13D, Erlangen, Germany). Data on four cerebral small vessel disease features are available, *i.e.*, total brain parenchyma volume, white matter hyperintensity volume, and presence of lacunar infarcts and cerebral microbleeds^35^. (Supplementary Information). In *dataset 2*, subjects were studied with a 3 T MRI scanner (Siemens Magnetom Skyra fit (Siemens Healthcare, Erlangen, Germany). Subjects were positioned supine inside the MRI and the head was immobilized with foam inserts on top of a radiofrequency probe. For signal acquisition, a 20-channel Head/Neck coil was used. After taking brain morphology scans, ^1^H-MRS spectrum was measured from a 20 × 30 × 30 mm voxel in the occipital lobe using PRESS sequence with 176 water-suppressed signal averages and 4 signal averages without water suppression. The occipital lobe was selected due to its reliability for spectral acquisition^36^. The voxel was carefully placed in the center of the occipital lobe, making sure that the margins of the voxel would not be close to the skull or the cerebellum (Fig. 1a). The measurement lasted 10 min. The sequence parameters were repetition time = 3000, echo time = 35 ms, bandwidth = 2000 Hz. Water suppression was performed using the CHESS method. Figure 1b shows a representative example of a fitted spectrum.

### Quantification of ^1^H-MRS data

Eddy current corrected ^1^H-MRS data were analyzed using LCModel (Version 6.3-1N)^37^. Water scaling was performed and the analysis window was set from 4.0 to 0.2 ppm. A simulated basis set^38^ was used on the acquired data to obtain the brain total NAA concentrations. Brain tissue fractions (gray matter, white matter, cerebrospinal fluid) were derived for more accurate estimation of the concentration of water which was used as an internal reference^39^. Subsequently, the metabolite/water ratio was converted to concentrations in mmol/L.

### Plasma NAA measurement

Plasma NAA concentrations were measured by HPLC–MS–MS and sample derivatization; the method has been validated in compliance with EMA guidelines and previously described in detail by Campi et al.^40^. In brief, plasma samples were thawed at room temperature and a 100 μL aliquot was added with 300 μL of a freshly prepared daily precipitation solution containing acetonitrile, formic acid 1% (V%), and internal standard. The resulting suspensions were vortexed, centrifuged, and 300 μL of the supernatants dried under a gentle stream of nitrogen. The dry residues were submitted to Fischer esterification reaction, and drying again under a nitrogen stream. The dry residues were reconstituted with 100 μL of ACN/H2O (20/80; V/V), and put into a 96 wells plate for the HPLC–MS–MS analysis. The injection volume of samples and calibrators was 5 μL. Calibrators were prepared by serial dilution in water at the following concentrations: 0.7813 (L1), 1.563 (L2), 3.125 (L3), 6.250 (L4), 12.50 (L5), 25.00 (L6), 50.00 (L7), 100.0 ng/mL (L8), 200.0 ng/mL (L9) to build calibration curve. The equation of that curve was used to convert the readings (Area analyte/Area Internal Standard) of the unknown samples into concentration.

### Cognitive function

Cognitive performance was assessed in *dataset 1* using a concise neuropsychological test battery^34^. Standardized test scores were calculated for the three cognitive domains: verbal memory, processing speed and executive function. We evaluated verbal memory with the Verbal Learning Test; processing speed with the Stroop Color-Word Test Part I and II, Concept Shifting Test Part A and B, and Letter-Digit Substitution Test; and executive function with the Stroop Color-Word Test Part III and Concept Shifting Test Part C^41^. These tests are presented in more detail in the Supplementary Information.

### Statistical analysis

Data are given as mean ± SD. Because plasma NAA concentrations had a skewed distribution, they were summarized as median [interquartile range IQR], and logarithmically transformed for use in parametric statistical tests. Group values were compared by the Mann–Whitney U-test. Linear and logistic regression analysis were used to evaluate the association between log-transformed circulating NAA levels (main exposure) and central NAA levels (main outcome dataset 2), cerebral small vessel disease features and domain-specific cognitive performance (main outcomes dataset 1). Data analyses were performed using JMP® 7.0 (SAS Institute Inc., 2007); a *p* value < 0.05 was considered statistically significant in two-sided tests.

## Results

### Circulating NAA, cognitive function and cerebral small vessel disease

In *Dataset 1*, we evaluated whether circulating NAA levels correlate with total brain parenchyma volume, white matter hyperintensity volume, and presence of lacunes and microbleeds using three statistical models: unadjusted, and adjusted for several potential confounders. The adjusted models showed that circulating NAA did not correlate with any feature of cerebral small vessel disease (Table 3). Similarly, whereas in univariate analysis circulating NAA levels were associated inversely with the speed in information processing and the executive function score, these associations were lost after accounting for confounders (Table 4). We then divided the group into quartiles of circulating NAA (Table 5). Because we noticed that cognitive functions and the features of cerebral small vessel disease varied slightly across quartiles of circulating NAA, we also tested whether there were any significant correlations between circulating NAA and these features when comparing the lowest to the higher quartiles of NAA. Once again, we did not detect any significant correlations between circulating NAA and the features of cognitive function and cerebral small vessel disease (Tables 6, 7).

**Table 3:** Associations between serum N-acetylaspartate levels (per higher standard deviation) and cerebral small vessel disease features.

	Total brain parenchyma volume (per + 1 SD)	White matter hyperintensity volume (per + 1 SD)^#^	Lacunes (yes/no)	Microbleeds (yes/no)
	β (95% confidence interval)		Odds ratios (95% confidence interval)	
Model 1	− 7 (− 16; 2)	− 0.01 (− 0.02; 0.00)	1.15 (0.83; 1.59)	0.96 (0.74; 1.24)
Model 2	− 6 (− 14; 3)	− 0.01 (− 0.02; 0.01)	1.05 (0.74; 1.47)	0.93 (0.71; 1.22)
Model 3	− 5 (− 15; 4)	− 0.00 (− 0.01; 0.01)	1.04 (0.74; 1.48)	0.97 (0.75; 1.27)

Model 1: unadjusted; Model 2: adjusted for age, sex, education and glucose metabolism status; Model 3: Model 2 + for prior cardiovascular disease, hypertension, smoking and body mass index.

Data available in n = 533.

^#^White matter hyperintensity volume was log-transformed.

**Table 4:** Associations serum N-acetylaspartate levels (per higher standard deviation) and domain-specific cognitive performance.

	Verbal memory Z-score (per + 1 SD)	Information processing speed Z-score (per + 1 SD)	Executive function Z-score (per + 1 SD)
	β (95% confidence interval)		
Model 1	− 0.03 (− 0.10; 0.03)	− 0.06 (− 0.11; − 0.01)	− 0.07 (− 0.12; − 0.02)
Model 2	0.00 (− 0.06; 0.06)	− 0.02 (− 0.07; 0.02)	− 0.04 (− 0.09; 0.01)
Model 3	− 0.01 (− 0.07; 0.05)	− 0.03 (− 0.07; 0.02)	− 0.04 (− 0.09; 0.01)

Model 1: unadjusted; Model 2: adjusted for age, sex, education and glucose metabolism status; Model 3: additionally adjusted for prior cardiovascular disease, hypertension, smoking and body mass index.

*Data available in n = 824.

**Table 5:** Subject characteristics by NAA quartiles.

	Quartile 1 NAA < 59.7 ng/ml	Quartile 2 NAA 59.7–70.3 ng/ml	Quartile 3 NAA 70.3–83.0 ng/ml	Quartile 4 NAA > 83.0 ng/ml
Age, years	57.6 ± 8.7	59.5 ± 8.2	60.3 ± 7.9	61.1 ± 7.6
Men, %	53.1	60.8	49.5	36.2
**Education**				
Low, %	29.0	33.8	31.1	36.7
Intermediate, %	29.0	32.8	31.6	24.6
High, %	42.0	33.3	37.4	38.6
**Glucose metabolism status**				
Normal glucose metabolism, %	44.9	49.9	53.9	63.3
Prediabetes, %	15.5	17.7	17.5	14.0
Type 2 diabetes, %	37.2	33.3	28.2	21.7
Other type diabetes, %	2.4	0.0	0.5	1.0
Body mass index, kg/m^2^	27.7 ± 4.3	27.5 ± 4.4	27.2 ± 4.0	25.9 ± 4.9
Current smokers, %	16.4	13.2	9.2	10.1
Systolic blood pressure, mmHg	136 ± 18	136 ± 17	134 ± 17	132 ± 19
Diastolic blood pressure, mmHg	77 ± 9	77 ± 10	76 ± 10	75 ± 10
Hypertension, %	63.1	59.3	57.6	47.3
Antihypertensive medication use, %	44.0	42.6	38.3	34.3
Prior cardiovascular disease, %	15.9	17.6	10.7	18.8
Features of cerebral small vessel disease*				
Total brain parenchyma volume, ml	1146 ± 109	1139 ± 114	1135 ± 115	1127 ± 108
White matter hyperintensity volume, median ml (IQR)	0.2 [0.1–0.6]	0.2 [0.1–0.7]	0.2 [0.1–0.7]	0.3 [0.1–0.7]
Cerebral microbleeds, %	11.2	11.8	15.4	9.7
Lacunar infarcts, %	5.1	8.7	4.1	7.5
**Cognitive function**				
Verbal memory, mean Z-score (SD)	0.19 ± 0.94	0.10 ± 0.84	0.15 ± 0.87	0.10 ± 0.95
Information processing speed, mean Z-score (SD)	0.08 ± 0.71	0.01 ± 0.76	0.08 ± 0.75	-0.05 ± 0.75
Executive function, mean Z-score (SD)	0.12 ± 0.68	0.09 ± 0.76	0.03 ± 0.75	-0.00 ± 0.78

*Data available in n = 533. NAA = serum N-acetylaspartate.

**Table 6:** Associations between serum N-acetylaspartate levels (lowest quartile *vs* highest quartile; highest quartile served as reference) and cerebral small vessel disease features.

	Total brain parenchyma volume (per + 1 SD)	White matter hyperintensity volume (per + 1 SD)^#^	Lacunes (yes/no)	Microbleeds (yes/no)
	β (95% confidence interval)		Odds ratios (95% confidence interval)	
Model 1	19 (− 8; 46)	0.02 (− 0.00; 0.05)	0.69 (0.25; 1.81)	1.18 (0.54; 2.59)
Model 2	11 (− 15; 36)	0.01 (− 0.03; 0.04)	0.83 (0.29; 2.33)	1.21 (0.54; 2.72)
Model 3	11 (− 16; 37)	0.01 (− 0.06; 0.04)	0.80 (0.28; 2.32)	1.09 (0.47; 2.53)

Model 1: unadjusted; Model 2: adjusted for age, sex, education and glucose metabolism status; Model 3: Model 2 + for prior cardiovascular disease, hypertension, smoking and body mass index.

Data available in n = 533.

^#^White matter hyperintensity volume was log-transformed.

**Table 7:** Associations between serum N-acetylaspartate levels (lowest quartile *vs* highest quartile; highest quartile served as reference) and domain-specific cognitive performance.

	Verbal memory Z-score (per + 1 SD)	Information processing speed Z-score (per + 1 SD)	Executive function Z-score (per + 1 SD)
	β (95% confidence interval)		
Model 1	0.10 (− 0.08; 0.27)	0.12 (− 0.02; − 0.27)	0.13 (− 0.02; 0.27)
Model 2	0.01 (− 0.16; 0.17)	0.01 (− 0.12; 0.13)	0.04 (− 0.10; 0.17)
Model 3	0.03 (− 0.15; 0.19)	0.02 (− 0.11; 0.14)	0.04 (− 0.10; 0.17)

Model 1: unadjusted; Model 2: adjusted for age, sex, education and glucose metabolism status; Model 3: additionally adjusted for prior cardiovascular disease, hypertension, smoking and body mass index.

Data available in n = 824.

In the whole dataset, circulating NAA levels correlated positively with age (*rho* = 0.16, *p* < 0.0001), and inversely with BMI (*rho* = − 0.18, *p* < 0.0001) and HbA_1c_ (*rho* = − 0.12, *p* = 0.001).

### Central and circulating NAA

*Dataset 2* included obese and lean individuals, who were well-matched for sex and age. Along with marked differences in adiposity measures, obese subjects also had higher prevalence of impaired glucose tolerance and T2D and thus higher HbA_1c_, higher mean arterial blood pressure, C-reactive protein, and triglycerides (Table 2). Central NAA and total NAA concentrations were significantly lower in the patients with obesity compared to the lean controls (Table 2). On the contrary, circulating NAA levels were not different between the two groups.

**Figure 2:**
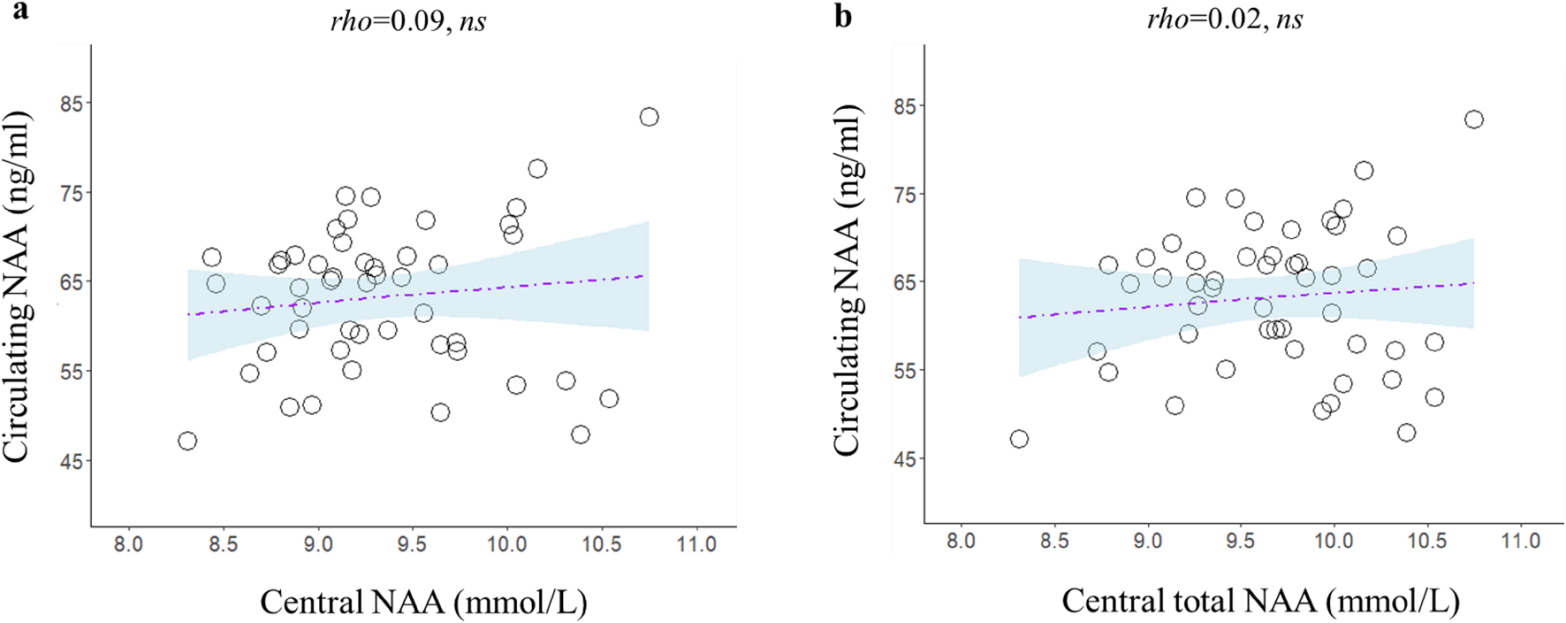
Relationship between brain NAA (**a**) and brain total NAA (**b**) and circulating NAA levels.

In univariate analysis, there was no significant correlation between central NAA or central total NAA and circulating NAA concentrations (Fig. 2).

## Discussion

Despite recent, major progress in the neurological field and a better understanding of the role of the brain in the orchestration of whole-body metabolism^42–49^, much remains to be investigated in human brain metabolism. Molecules that originate primarily from the brain but spill over into the blood in quantifiable levels represent good candidate biomarkers for assessing cerebral function. One such molecule is NAA, an aminoacid mainly produced in neurons and secondarily in astrocytes after hydrolysis of the glutamate moiety from NAAG. Several lines of research have shown that cerebral NAA levels are decreased in several cerebral diseases comprising Alzheimer’s, Parkinson and Huntngton’s disease^8,11,15^. However, thus far it was not known whether circulating NAA levels reflect brain function. Our study shows that, in a large sample of subjects from the general population, circulating NAA does not correlate with any features of cerebral small vessel disease or cognitive function.

First, to test whether circulating NAA reflects brain function, we chose to study functional (cognition) and structural (small vessel disease) characteristics that have been previously linked with central NAA levels. There is a large body of evidence showing that central metabolites associate with cognitive function in both healthy subjects and in patients with neurological damage, even though other studies have shown no correlation between central NAA levels and cognition (as reviewed by Ross and Sachdev^26^). In healthy subjects, central NAA has been positively related to performance on intelligence tests working memory tasks in children and adults^50–52^, and with executive-attentional cognitive tasks and facial recognition in the elderly^53–55^. As plausible explanations for these findings, it has been proposed that higher central NAA may be associated with greater neuronal density, better metabolic efficiency, or increased synaptic connectivity (*i.e.* the ensemble of direct chemical and electrical connections between neurons^56^)^26^. However, the existing literature on the relationship between central NAA levels and cognition is not unanimous. For instance, Patel and colleagues reviewed 14 studies and reported that the average correlation between NAA and various measures of cognitive ability was 0.39, with a relatively high standard deviation of 0.23, suggesting inhomogeneous effects across studies^27^.

Cerebral small vessel disease is a major cause of cognitive impairment and dementia^31^. In a brain ^1^H-MRS study, Nitkunan et al*.* showed that, in subjects with cerebral small vessel disease, central NAA concentrations are reduced and negatively correlated to the lesion volume^28^.

In the current study circulating NAA was associated neither with cognitive function nor with features of small vessel disease. On the contrary, we confirmed the previously described correlations of circulating NAA with age, BMI and HbA_1c_^57^. Based on these findings in a large number of subjects, we hypothesized that the lack of association between circulating NAA and functional or structural characteristics of the brain could be explained by assuming that circulating NAA does not reflect central NAA levels.

Indeed, in the small sample of subjects who were studied with brain ^1^H-MRS, contemporaneous assessment of central and circulating NAA levels showed that the two measurements were unrelated (Fig. 2). Even though our study cannot assess the reason(s) for the discrepancy between central and circulating NAA values, there are several steps where this mismatch could occur, from the spillover from the brain into the bloodstream or from differences in NAA removal from the plasma among different subjects (or a combination thereof)^4^.

Strengths of the present study are the large number of subjects studied in *dataset 1* and the direct comparison of central and peripheral NAA in the same subjects in *dataset 2*. Our study also has limitations. First, the sample of subjects in whom brain ^1^H-MRS was performed was rather small; future studies are needed to replicate the present findings. In addition, ^1^H-MRS was acquired only from the occipital lobe, thus generalization of our ^1^H-MRS findings to the whole-brain level should be made with caution. Finally, in *dataset 1* the acquisition of the fasting samples for the measurement of circulating NAA was not synchronous with the MRI or the cognitive function evaluation (there was a time lag of weeks). Considering the dynamic nature of central NAA, this might have affected the present results; on the other hand, it seems implausible that either cognitive function or cerebral small vessel disease would change in such a short time period in otherwise neurologically healthy subjects.

In conclusion, the current study shows that circulating NAA does not predict presence or severity of small vessel disease or cognitive dysfunction in a large dataset of neurologically healthy individuals. Thus, alterations in circulating NAA levels do not seem of relevant clinical use in predicting—at an early stage—either cognitive decline, or cerebral SVD. However, since the present datasets consisted only of subjects with no previous and current neurologic diagnoses, further research is necessary to assess whether circulating NAA concentrations reflect central NAA levels and/or cognition in patients. Also, whether circulating NAA is merely a “spillover” marker or whether it elicits any cross-talk effects between the brain and the periphery warrants investigation.

## Supplementary Information

Supplementary Information.

## Data Availability

The datasets used and/or analyzed during the current study are available from the corresponding author on reasonable request.

## Abbreviations

ASPA: Aspartoacylase
BMI: Body mass index
CNS: Central nervous system
HPLC: High-performance liquid chromatography
MRI: Magnetic resonance imaging
MRS: Magnetic resonance spectroscopy
NAA: N-acetylaspartate
NAAG: N-acetyl aspartate glutamate
SVD: Small vessel disease
T2D: Type 2 diabetes
TNAA: Total NAA
