# Supplementary material for: Circulating N-Acetylaspartate does not track brain NAA concentrations, cognitive function or features of small vessel disease in humans
Source: Sci Rep. 2022 Jul 7;12:11530. doi: 10.1038/s41598-022-15670-0 (PMC9262942; doi:10.1038/s41598-022-15670-0)
Supplement: Supplementary file 1 — Supplementary Information. [file 41598_2022_15670_MOESM1_ESM.docx]

**Supplementary Material**

**Description of the MRI and cognitive measurements done in The Maastricht Study (*Dataset 1*)**

**Brain magnetic resonance imaging measures**

Brain magnetic resonance imaging (MRI) was performed on a 3T MRI scanner (Siemens Magnetom Prisma-fit Syngo MR D13D, Erlangen, Germany) by use of a 64-element head/neck coil for parallel imaging. The MRI protocol consisted of a 3D T_1_-weighted magnetization prepared rapid acquisition gradient echo (MPRAGE) sequence (TR/TI/TE 2300/900/2.98 ms, 176 slices, 256×240 matrix size, 1.00 mm cubic voxel size); a fluid-attenuated inversion recovery (FLAIR) sequence (TR/TI/TE 5000/1800/394 ms, 176 slices, 512×512 matrix size, 0.49×0.49×1.00 mm voxel size); a combined proton density (PD) and T_2_-weighted turbo spin echo (TSE) pulse sequence (TR/TE1/TE2 3200/9.4/94 ms, 30 slices, 640×540 matrix size, 0.36×0.36×4.00 mm voxel size); and a susceptibility-weighted imaging (SWI) sequence (TR/TE 28/20 ms, 144 slices, 384×312 matrix size, 0.57×0.57×1.00 mm voxel size).

Contra-indications for MRI assessments were the presence of a cardiac pacemaker or implantable cardioverter-defibrillator, neurostimulator, non-detachable insulin pump, metallic vascular clips or stents in the head, cochlear implant, metal-containing intra-uterine device, metal splinters or shrapnel, dentures with magnetic clip, an inside bracket, pregnancy, epilepsy, and claustrophobia.

T_1_-weighted images and FLAIR images were analyzed by use of an ISO-13485:2012 certified, automated method (which included visual inspection). T_1_-weighted images were segmented into grey matter, white matter and cerebrospinal fluid volumes (1 voxel = 1.00 mm^3^ = 0.001 ml). Intracranial volume was calculated as the sum of grey matter, white matter (including white matter hyperintensity volume) and cerebrospinal fluid volumes. Total brain parenchyma volume was calculated as the sum of grey and white matter volumes. White matter hyperintensities identified were summed to assess total white matter hyperintensities burden in milliliter. For analyses, total brain parenchyma volume and white matter hyperintensities were expressed as percentage of total intracranial volume. Lacunar infarcts were defined as focal brain parenchyma defects of ≥3 mm and <15 mm in size with a similar signal intensity as cerebrospinal fluid on all sequences and a hyperintense rim on T_2_ and FLAIR images.^1^ Cerebral microbleeds were rated on T_2_-weighted and SWI images by use of the Microbleed Anatomical Rating Scale, and were defined as focal lesions of ≥2 mm and ≤10 mm in size with a hypointense signal. The presence of lacunar infarcts and cerebral microbleeds was rated manually by three neuroradiologists. The two-way mixed effects, consistency, intraclass correlation coefficients for the three raters based on 50 randomly selected scans were 0.84 (95% confidence interval 0.74; 0.91) and 0.83 (0.72; 0.90) for the presence of lacunar infarcts and cerebral microbleeds, respectively.

**Description of the individual cognitive tests used in the present study (*Dataset 1*)**

**Verbal Learning Test:**

Fifteen unrelated, monosyllabic, words were presented on a computer screen in five subsequent trials. After each trial, participants were instructed to recall as many words as possible in any order. Twenty minutes after the last trial, participants were asked again to reproduce the words. Outcomes recorded included the total number of words correctly recalled over the five trials (total immediate recall) and the number of correctly recalled words during delayed recall (delayed recall).

**Stroop Color-Word Test:**

In this test, which consisted of three parts, participants were firstly asked to read aloud color names (i.e. red, blue, yellow, and green) that were printed in black ink (Part I). Secondly, they were instructed to name solid color patches (Part II). Finally, participants had to name the ink color of color names that were printed in an incongruent color (e.g. participants were asked to say red when the word yellow was printed in red) (Part III). The time needed to complete Part III was adjusted for the average time needed to complete Part I and II.

**Concept Shifting Test:**

This test, a modification of the Trailing Making Test, consisted of four subtasks. During each subtask, participants were shown 16 small circles aligned along a larger imaginary circle. The small circles contained (a combination of) digits, letters, or were empty. Participants were instructed to cross-out as quickly as possible the digits in ascending order (Part A), the letters in alphabetic order (Part B), and the letters and digits in alternating order (Part C). Thereafter, participants were asked to cross-out empty circles in a clockwise fashion in two consecutive trials (Part 0). In this way, test results could be accounted for basic motor speed. The time needed to complete subtasks A and B was adjusted for the average time needed to complete Part 0, the time needed to completed Part C for the average time of Part A and B.

**Letter-Digit Substitution Test:**

Participants were requested to match digits to letters according to a given key. This key included the numbers 1 to 9, each paired with a different letter. The outcome of interest was the number of correct substitutions within 90 seconds.

**References**

1. Wardlaw JM, Smith EE, Biessels GJ, et al. Neuroimaging standards for research into small vessel disease and its contribution to ageing and neurodegeneration. *Lancet Neurol* 2013;12(8):822-38. doi: 10.1016/S1474-4422(13)70124-8 [published Online First: 2013/07/23]
